# HCV coinfection contributes to HIV pathogenesis by increasing immune exhaustion in CD8 T-cells

**DOI:** 10.1371/journal.pone.0173943

**Published:** 2017-03-21

**Authors:** Norma Rallón, Marcial García, Javier García-Samaniego, Noelia Rodríguez, Alfonso Cabello, Clara Restrepo, Beatriz Álvarez, Rosa García, Miguel Górgolas, José M. Benito

**Affiliations:** 1 IIS-Fundación Jiménez Díaz, UAM, Madrid, Spain; 2 Hospital Universitario Rey Juan Carlos, Móstoles, Spain; 3 Hospital Universitario Carlos III-La Paz, Madrid, Spain; 4 Hospital Universitario Fundación Jiménez Díaz, Madrid, Spain; University of Hawaii System, UNITED STATES

## Abstract

**Background:**

There are several contributors to HIV-pathogenesis or insufficient control of the infection. However, whether HIV/HCV-coinfected population exhibits worst evolution of HIV-pathogenesis remains unclear. Recently, some markers of immune exhaustion have been proposed as preferentially upregulated on T-cells during HIV-infection. Herein, we have analyzed T-cell exhaustion together with several other contributors to HIV-pathogenesis that could be affected by HCV-coinfection.

**Patients and methods:**

Ninety-six patients with chronic HIV-infection (60 HIV-monoinfected and 36 HIV/HCV-coinfected), and 20 healthy controls were included in the study. All patients were untreated for both infections. Several CD4 and CD8 T-cell subsets involved in HIV-pathogenesis were investigated. Non-parametric tests were used to establish differences between groups and associations between variables. Multivariate linear regression was used to ascertain the variables independently associated with CD4 counts.

**Results:**

HIV-patients presented significant differences compared to healthy controls in most of the parameters analyzed. Both HIV and HIV/HCV groups were comparable in terms of age, CD4 counts and HIV-viremia. Compared to HIV group, HIV/HCV group presented significantly higher levels of exhaustion (Tim3^+^PD1^-^ subset) in total CD8^+^ T-cells (p = 0.003), and higher levels of exhaustion in CD8^+^HLADR^+^CD38^+^ (p = 0.04), CD8^+^HLADR^-^CD38^+^ (p = 0.009) and CD8^+^HLADR^-^CD38^-^ (p = 0.006) subsets of CD8^+^ T-cells. Interestingly these differences were maintained after adjusting by CD4 counts and HIV-viremia.

**Conclusions:**

We show a significant impact of HCV-coinfection on CD8 T-cells exhaustion, an important parameter associated with CD8 T-cell dysfunction in the setting of chronic HIV-infection. The relevance of this phenomenon on immunological and/or clinical HIV progression prompts HCV treatment to improve management of coinfected patients.

## Introduction

Human immunodeficiency virus (HIV) and hepatitis C virus (HCV) are two of the most relevant persistent infections afflicting the human population, with 37 and 150 million chronically infected individuals worldwide respectively [1]. Coinfection with both viruses is common due to the existence of shared transmission routes [2] and as much as 20–30% of HIV positive patients are coinfected with HCV [1]. Since both viruses interfere with host immune response [3, 4] and interact at the molecular level [5, 6], coinfection poses a formidable challenge to host immune system. As a consequence of this, each virus can impact on the clinical course of the other. In fact, the negative impact of HIV infection on hepatitis C pathogenesis is well described. HIV/HCV coinfection has been associated with lower rates of spontaneous HCV clearance, higher HCV viral load, accelerated liver disease progression and reduced response to interferon-based HCV therapy [1]. Since the introduction of highly active antiretroviral therapy (HAART), the clinical prognosis for HIV patients has markedly improved and, as a result, HCV infection is now a leading cause of morbidity and mortality in HIV/HCV coinfected individuals [7]. This coinfection is probably also associated with more rapid progression of HIV disease, but data on the impact of HCV coinfection on HIV disease progression and mortality are controversial [8–11].

Immune defects caused by HCV infection could impact on different markers of HIV disease progression, although this aspect of HIV/HCV interaction has been less studied, with most previous studies addressing only a few markers of HIV progression. Among the different markers, T-cell activation and T-cell apoptosis have been addressed in different studies with discordant results. Thus, some studies have found higher levels of T-cell activation [12–14] and of T-cell apoptosis [14–16] in HCV/HIV coinfection compared to HIV monoinfection, but not others [16–18]. Much more scarce are studies addressing the effect of HCV on other important markers of HIV pathogenesis, such as senescence, cell turnover and maturation stage of T cells [19]. Regarding T-cell exhaustion, which is an important mechanism of T-cell dysfunction in the setting of chronic viral infections [20], it has been explored in HIV [21–24] and HCV [25] infections, as well as in the setting of HIV/HCV coinfection [14, 26, 27]. In this setting it has been shown that HIV increases the level of exhaustion of HCV-specific T cells [26], but no study so far has evaluated the impact of HCV presence on exhaustion of HIV-specific T cells in the HIV/HCV coinfected population.

In the present study, we have tested the effect of HCV infection on several aspects of HIV disease pathogenesis, performing an in-depth analysis of several markers associated with HIV progression in a large cohort of individuals including HIV/HCV coinfected and HIV monoinfected patients. The demonstration of HCV-induced impairment of any of these markers, will support early treatment of HCV in order to attenuate or halt not only the accelerated liver injury observed in HIV/HCV coinfected patients, but also the deleterious effect of HCV on HIV disease progression.

## Patients and methods

### Study population

This cross-sectional study included a total of 116 subjects, distributed as follows: 60 patients with chronic HIV infection (HIV group), 36 patients with chronic HIV infection and coinfected with HCV (HIV/HCV goup), and 20 age and sex-matched HIV and HCV seronegative healthy controls. All patients were naïve for anti-HCV treatment and for antiretroviral therapy (ART) at the moment of study. To participate in the study, written informed consent was obtained from all individuals, and the study protocol was evaluated and approved by the Ethical Committee of Hospital Carlos III-La Paz.

### Cell samples

All analyses were done in cryopreserved peripheral blood mononuclear cells (PBMCs). EDTA-anticoagulated blood was obtained by venipuncture; PBMCs were immediately isolated by density gradient centrifugation using Ficoll-Hypaque (Sigma Chemical Co., St. Louis, MO) and frozen in FCS plus 10% DMSO. Viability of thawed PBMCs was always greater than 85%.

### Immunophenotypic analysis

We used a comprehensive approach of simultaneous measurement of different T-cells parameters involved in HIV pathogenesis. For CD8 T cells the next parameters were analysed: level of exhaustion (measuring PD1 and Tim3 expression), and level of activation (measuring CD38 and HLADR expression). For CD4 T cells the next parameters were evaluated: naïve/memory subsets (using CD45RA expression), recent thymic emigrants (RTE, using CD31 expression), cell turnover (using Ki67 expression), apoptosis (using CD95 expression), senescence (using CD57 expression), and exhaustion (using PD1 and Tim3 expression). All parameters were evaluated using multiparameter flow cytometry. PBMCs from each patient were stained with proper antibodies according to the different parameters evaluated. A detailed description of staining conditions is given as S1 Text, a complete list of all monoclonal antibodies and fluorochromes used in the study is shown in S1 Table and a representative flow cytometry experiment illustrating the gating strategies for CD4 and CD8 cell subsets analyses is shown in S1 Fig.

### Statistical analysis

The main characteristics of the study population, and the different parameters evaluated are expressed as median [interquartile range]. Comparisons between groups were done using Mann-Whitney U-test. Correlations between quantitative parameters were explored using Pearson correlation coefficient, and between qualitative variables using the Chi-square test or Fishers’s exact test as appropriate. All statistical analyses were performed using the SPSS software version 15 (SPSS Inc., Chicago, IL, USA). All p-values were two-tailed, and were considered as significant only when lower than 0.05.

## Results

### Patient’s characteristics

The main characteristics of patients at the time of inclusion in the study are shown in Table 1. All patients were naïve for both anti-HCV and ART at the moment of inclusion in the study. There were no significant differences between HIV and HIV/HCV groups in terms of age, CD4 counts, and plasma HIV-RNA load. However, proportion of males was significantly higher in HIV group and the distribution of risk factors for HIV acquisition was also significantly different. MSM risk factor was the most prevalent in HIV group (76%), whereas IDU risk factor was the most prevalent in HIV/HCV group (81%).

**Table 1:** Characteristics of patients included in the study.

Characteristic	HIV group(n = 60)	HIV/HCV group(n = 36)	p-value
Median age (years)	46 [40–51]	48 [45–51]	0.61
Gender (% of males)	93%	69%	**0.002**
Median CD4 count (cells/μl)	436 [302–702]	411 [321–546]	0.53
Median HIV-RNA (log copies/mL)	4.3 [3.9–4.7]	4.1 [3.4–4.6]	0.19
Median HCV-RNA (log copies/mL)	-	6.1 [5.0–6.7]	-
Group risk for HIV infection			**<0.0001**
Intravenous drug user (IDU)	2%	81%	
Heterosexual	22%	10%	
Male sex with male (MSM)	76%	9%	

### Subsets of CD4 T-cells

**Fig 1:**
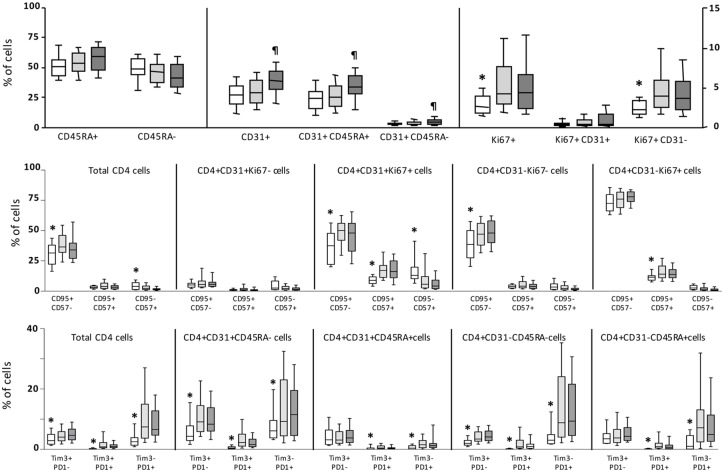
Levels of CD4 T-cell subsets. Box-plot graphs showing the levels of CD4 T-cell subsets in healthy controls (clear boxes), HIV (light grey boxes) and HIV/HCV (dark grey boxes) patients. Upper graph shows levels of different CD4 subsets on the basis of CD45RA, CD31 and Ki67 expression. Right vertical axis applies only for subsets defined by Ki67 and CD31. Middle graph shows levels of apoptosis (CD95) and senescence (CD57) on different subsets of CD4 cells; and lower graph the levels of exhaustion (PD1 and Tim3 markers) on different subsets of CD4 cells. Statistically significant differences between healthy controls and all patients are marked by an asterisk; and differences between the two groups of patients by ¶ symbol.

Different subsets of CD4 T-cells related to several aspects of CD4 homeostasis and function were evaluated, including naïve/memory subsets, recent thymic emigrants (RTE), cell turnover, apoptosis, senescence and exhaustion with no significant differences found between groups of patients. However, several of these subsets were significantly altered in the whole population of patients compared to healthy controls (Fig 1). Cell turnover (Ki67+ cells) was significantly increased, mainly in CD31- (non RTE) cells. Apoptosis was also significantly increased in total CD4 cells as well as in different subsets defined by CD31 and Ki67 markers. Levels of senescence (CD57 marker) were much lower than levels of apoptosis, although the subset of cells co-expressing both apoptosis and senescence markers (CD95+CD57+ cells) were significantly increased in proliferating (both CD31+Ki67+ and CD31-Ki67+) cells from patients compared to cells from healthy controls. Cell exhaustion was also significantly increased in the whole population of patients compared to healthy controls. Both PD1 and Tim3 were significantly increased in total CD4 cells as well as in all subsets defined by CD31 and CD45RA markers. Overall, levels of PD1 were higher than levels of Tim3. This increase in exhaustion was highest in memory (CD45RA-) cells and lowest in naïve RTE (CD31+CD45RA+) cells.

### Subsets of CD8 T-cells

**Fig 2:**
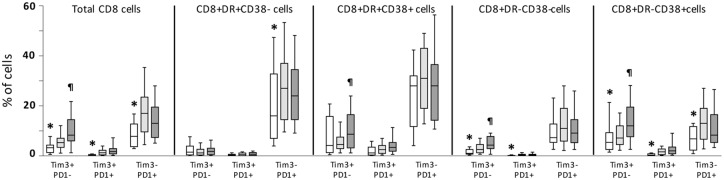
Levels of exhaustion markers on CD8 T-cells. Box-plot graph showing the level of exhaustion (PD1 and Tim3 markers) on different subsets of CD8 T-cells in healthy controls (clear boxes), HIV (light grey boxes) and HIV/HCV (dark grey boxes) patients. Statistically significant differences between healthy controls and all patients are marked by an asterisk; and differences between the two groups of patients by symbol.

Activation and exhaustion of CD8 T-cells were evaluated in the whole study population. Activation was significantly increased (CD38+HLADR+ and CD38+HLADR- subsets) in the whole population of patients compared to healthy controls, and levels were similar in both groups of patients (data not shown). Exhaustion was analyzed in total CD8 T-cells and in different subsets defined by CD38 and HLADR markers (Fig 2). In the whole population of patients, exhaustion levels (both PD1 and Tim3 markers) were significantly increased compared to healthy controls. Of note, exhaustion was increased in activated and non-activated subsets of CD8 T-cells, with different patterns: both PD1 and Tim3 markers were increased in CD38+HLADR- subset of CD8 cells, Tim3 expression was increased in non-activated (CD38-HLADR-) subset, and PD1 was increased in CD38-HLADR+ subset. Interestingly, when comparing HIV and HIV/HCV groups of patients, a significant increase in exhaustion was observed in total CD8 T-cells as well as in several subsets defined by CD38 and HLADR expression in HIV/HCV compared to HIV patients (Fig 2).

### Associations of T-cell subsets with CD4 counts, plasma HIV-RNA load, and presence of HCV coinfection

In the whole population of patients both bivariate (Pearson correlation) and multivariate (linear regression) analysis were performed to test potential associations of the different T-cell subsets with immunological (CD4 counts) and virological (plasma HIV-RNA load; HIV-pVL) markers of HIV disease as well as with presence of HCV coinfection. In the bivariate analysis, many of the T-cell subsets analyzed were inversely correlated with CD4 counts (S2 Table). Thus, exhaustion of both CD4 and CD8 T-cells; activation of CD8 T-cells; apoptosis of CD4; and turnover of CD4 cells were inversely and significantly correlated with CD4 counts. Moreover, several of these parameters were significantly and directly correlated with levels of HIV-pVL.

A multivariate linear regression analysis using CD4 counts, HIV-pVL, presence of HCV coinfection, drug abuse and gender as explanatory variables, and the different immune parameters as dependent variables, revealed that some parameters involved in HIV pathogenesis could be affected by HCV coinfection: a) exhaustion of CD4 T-cells was associated only with HIV-pVL; b) level of naïve RTE CD4 T-cells with presence of HCV coinfection; c) apoptosis and turnover of CD4 T-cells was associated with CD4 counts; d) activation (CD38+HLADR- subset) of CD8 T-cells was associated with HIV-pVL and almost significantly with presence of HCV coinfection; e) exhaustion of CD8 T-cells was associated with both presence of HCV coinfection and CD4 count (Table 2). Interestingly, when considering only the HIV/HCV coinfected group of patients and level of HCV-RNA load was included as another potential explanatory variable, the only parameter associated with exhaustion of CD8 T-cells was the HCV-RNA load (R = 0.46; ß ± SD = 2.18±0.90; p = 0.03).

**Table 2:** Multivariate linear regression models showing the association of CD4 counts, HIV-pVL and presence of HCV coinfection, with different T-cells subsets in the whole population of patients. Data shown is adjusted by gender and drug abuse.

		Explanatory variables ß±SD p-value		
T-cells subsets (dependent variable)	R of the model	CD4 count (cells/μL)	HIV- pVL (Log cop./mL)	HCV coinfection
**CD4 subsets**				
Tim3+PD1-	**0.25**	-0.01±0.02 p = 0.77	**1.36±0.59 p = 0.02**	0.11±1.94 p = 0.96
CD31+CD45RA+	**0.33**	0.003±0.005 p = 0.54	1.39±1.67 p = 0.41	**8.31±2.66 p = 0.003**
CD31-CD95+	**0.49**	**-0.02±0.004 p< 0.0001**	0.39±1.66 p = 0.82	-2.15±2.21 p = 0.33
CD31-Ki67+	**0.38**	**-0.004±0.001 p< 0.0001**	0.88±0.48 p = 0.07	-0.90±0.66 p = 0.18
**CD8 subsets**				
CD38+HLADR-	**0.48**	-0.1±0.006 p = 0.12	**10.2±2 p< 0.0001**	5.9±3.2 p = 0.06
Tim3+PD1-	**0.37**	**-0.006±0.002 p = 0.009**	0.54±0.91 p = 0.55	**2.6±1.2 p = 0.036**

In order to ascertain which of the many immune parameters analyzed were more related to HIV immunological progression, the association with CD4 counts was evaluated in a multivariate linear regression. In this analysis, HIV-pVL and T-cell subsets showing a significant association with CD4 counts were included as explanatory variables. Results of this analysis revealed that apoptosis of CD4+CD31- T-cells; HIV-pVL; turnover of CD4+CD31+ T-cells; and exhaustion of CD8 T-cells were significantly and independently associated with CD4 counts (Table 3).

**Table 3:** Linear regression model showing the parameters significantly and independently associated with CD4 counts in the whole population of patients.

	Percentage of variation explained by the model (R2)			
Variables in the model	Individual	Accumulated	Regression coefficient (ß±SD)	p-value
CD4+CD31-CD95+ (%)	0.260	**0.260**	-11.8±2.2	<0.0001
HIV pVL (log copies/mL)	0.147	**0.407**	-125.9±29.2	<0.0001
CD4+CD31+Ki67+ (%)	0.064	**0.471**	-56.2±20.2	0.007
CD8+PD1-Tim3+ (%)	0.029	**0.474**	-8.2±3.9	0.037

## Discussion

The present study was designed to evaluate the potential impact of coinfection with HCV virus on several markers of HIV disease progression. A large panel of T-cell markers were analyzed, some of them with well known influence on HIV progression (activation, apoptosis, cell turnover) [28], and others more recently described as involved in HIV pathogenesis, such as T-cell exhaustion [29]. Our data show that in untreated HIV infection, coinfection with HCV does impact on activation and exhaustion of CD8 T-cells, which are important parameters, associated with HIV pathogenesis [23, 24, 28]. Of note, exhaustion is also implicated in the dysfunction of CD8 T-cells in the setting of chronic HIV infection [30].

We analyzed a large panel of CD4 subsets and, as expected for untreated HIV-infected individuals, the majority of these subsets were altered as compared to healthy controls. Apoptosis and cell turnover of CD4 cells were increased, in agreement with previous reports [31, 32]. Interestingly, expression of apoptosis-associated marker CD95 was higher in proliferating (Ki67+) than in non-proliferating (Ki67-) CD4 cells. Although CD95 is a molecule involved in the extrinsic pathway of apoptosis and has a central role in HIV infection [33], it is up-regulated upon T-cell activation, and thus the higher expression we found on proliferating cells may be related to the activation status of this subset of cells. Interestingly, in a multivariate analysis, CD4 count was significantly and inversely associated with CD95 and Ki-67 expression on CD4 cells, suggesting a role for CD95 in cell depletion, and for cell turnover in the homeostasis of CD4 cells, as has been previously proposed [33, 34].

Exhaustion of CD4 cells was significantly increased in the whole population of patients in agreement with previous studies [24, 35–39]. Both PD1 and Tim-3 were increased in different subsets of CD4 cells [36, 38], with the lowest levels observed in RTEs and the highest in memory (CD45RA-) cells. Overall, PD1 expression was higher than Tim-3, suggesting different regulatory mechanisms as well as different correlations with functional impairment of T cells for these markers [29]. Coexpression of both markers was very low and likely is associated with higher levels of functional impairment [35]. Levels of CD4 exhaustion were directly correlated with plasma HIV viremia and not with CD4 counts, suggesting a direct role of HIV replication on CD4 exhaustion [35, 39].

Overall, and in contrast with some previous studies [14–16], the different parameters evaluated in CD4 T-cell were similar in both groups of studied patients. Thus, it seems that HCV coinfection does not affect the CD4 parameters involved in HIV pathogenesis in this study cohort. Interestingly, our results are in agreement with other more recent studies [17, 18, 27, 40]. As is evident, this subject is still controversial and needs further study. Probably the different characteristics of the studied cohorts (level of HIV-pVL, HIV treatment status, epidemiological characteristics of patients, and different assays used to measure the different immunological parameters) may account for these seemingly contradictory results.

Regarding the different parameters evaluated on CD8 T-cells, as potential contributors of HIV pathogenesis, both activation and exhaustion were influenced by HCV presence. Activation of CD8 cells is one of the main factors associated with CD4 depletion and HIV disease progression [41]. In agreement with this, we found significantly increased levels of activated subsets of CD8 cells in the whole population of patients that were directly correlated with HIV plasma viremia and inversely with CD4 counts. Moreover, activation (CD38+HLADR- subset) of CD8 T-cells was associated with presence of HCV coinfection in a multivariate analysis correcting by HIV-pVL and CD4 counts, drug abuse and gender. Of note, our results confirm what was reported by two studies: Kovacs et al., in woman coinfected with HIV/HCV [12]; and Gonzalez et al., in HIV/HCV coinfected patients on HAART [13]. Our results are also similar to what was reported by Feuth et al., on CD4 T-cells. They found a significantly higher level of activation on CD4 T-cells in HIV/HCV coinfected patients as compared to HCV or HIV monoinfection [14].

Exhaustion of CD8 cells (both PD1 and Tim-3 expression) was also significantly increased in the whole population of patients in agreement with previous reports [24, 35–39]. This increase was observed both in activated and in resting subsets of CD8 cells, with a different pattern depending on the activation status. Thus, in resting cells (CD38-HLADR- subset) only Tim-3 was significantly increased, whereas in activated cells (CD38+HLADR- subset) there was a significant increase of all three subsets defined by PD1 and Tim-3 markers. Interestingly, exhaustion was not significantly increased in CD38+HLADR+ CD8 cells, and only PD1 was up-regulated in CD38-HLADR+ cells. Therefore, these data suggests that although activation and exhaustion are linked phenomena, as has been previously proposed [37, 42], other additional mechanisms different from activation are likely involved in up-regulation of exhaustion in the setting of HIV and HCV/HIV infections. Our data also suggest that different mechanisms may be involved in regulation of PD1 and Tim-3 expression in agreement with previous reports [42].

Exhaustion of CD8 T-cells was significantly higher in HIV/HCV than in HIV patients. Of note, this increase was observed in both activated and resting CD8 T-cells. Only two previous studies have analyzed the effect of HCV coinfection on exhaustion of T-cells in HIV-infected patients [14, 27] with results along the same line to ours. Saha et al., reported the increase of exhaustion markers in a particular subset of T-cells (effector memory T cells) during hepatitis C virus/HIV coinfection as compared to hepatitis C virus or HIV monoinfection [27]. Feuth et al., suggest that HCV has a complementary role in exhaustion (PD-1) of CD4 T-cells in HIV/HCV coinfection [14]. Of note, ours is the first study reporting a significant impact of HCV coinfection on Tim-3 expression on total CD8 T-cells from untreated HIV/HCV coinfected patients. Interestingly, level of Tim-3 expression on CD8 cells was one the factors significantly and independently associated with CD4 counts in a multivariate analysis, suggesting an important role for CD8 exhaustion in the immunological progression of HIV infection. Thus, the higher levels of this marker observed in co-infected patients could impact on the rate of CD4 decline in untreated HIV patients. Further studies with a longitudinal design are necessary to confirm these interesting results.

In summary, our study shows that some parameters involved in HIV pathogenesis (activation and exhaustion of CD8 T-cells) are influenced by HCV coinfection. Of note, presence of HCV coinfection does significantly increase the levels of CD8 T-cell exhaustion. Since this is one of the parameters associated with CD4 counts, this suggests that presence of HCV coinfection could impact on HIV immunological progression, although follow up studies are necessary to confirm this hypothesis. Our results also reinforce the necessity of anti-HCV treatment in every patient in the setting of chronic HIV/HCV infection, irrespective of the stage of liver disease.

## Supporting information

S1 Text Staining conditions for immunophenotypic analysis. (DOC)

S1 Table Monoclonal antibodies and fluorochromes used in the study. Complete list of all monoclonal antibodies and fluorochromes used in the study.
(DOC)

S2 Table Pearson correlation coefficients (bivariate analysis) of different T-cell subsets with CD4 counts and with HIV pVL, in the whole population of patients. Only significant correlations are shown.
(DOC)

S1 Fig Representative examples of flow cytometry data and gating strategy. An initial gating was applied using forward (FSC) and side (SSC) scatter. From the population of lymphocytes a gate was placed to select CD4+ T-cells (staining panels 1 and 3) or CD8+ T-cells (staining panel 2). Then, the coexpression of CD45RA and CD31 and the coexpression of CD31 and Ki-67 were analyzed in CD4 T-cells and the coexpression of CD38 and HLADR was analyzed in CD8 T-cells. Lastly, expression of exhaustion markers PD1 and Tim3 was analyzed in different subsets of CD4 and CD8 T-cells, and the expression of CD95 and CD57 was analyzed in different subsets of CD4 T-cells.
(TIF)
