# Supplementary material for: HCV coinfection contributes to HIV pathogenesis by increasing immune exhaustion in CD8 T-cells
Source: PLoS One. 2017 Mar 21;12(3):e0173943. doi: 10.1371/journal.pone.0173943 (PMC5360268; doi:10.1371/journal.pone.0173943)
Supplement: S1 Text — (DOC) [file pone.0173943.s001.doc]

**S1 Text.**

*Staining conditions for immunophenotypic analysis*

Three different antibody panels were used for evaluation of the different CD4 and CD8 T cell subsets. Combinations of conjugated antibodies in the panels were as follows: Panel 1 included CD31-FITC, Tim3-PE, CD45RA-ECD, CD4-PercPVio700, PD1-biotin/streptavidin-PeCy7; Panel 2 included CD38-FITC, Tim3-PE, CD8-ECD, HLADR-PECy5, PD1-biotin/streptavidin-PeCy7; Panel 3 included CD31-FITC, Ki67-PE, CD57-PECF594, CD4-PercPVio700, CD95-PECy7.

A million of PBMC were washed with phosphate-buffered saline (PBS) and stained for surface markers by incubating with the appropriate antibody panel for 30 min at 4ºC. For antibody panels 1-2, cells were then washed with PBS, incubated with Streptavidin-PECy7 for 30 min at 4ºC, washed with PBS and resuspended in 250 µL of PBS for data acquisition. For antibody panel 3 cells were washed with PBS, resuspended in 1mL of Foxp3 fixation/permeabilization solution and incubated for 30 min at room temperature, and washed with 2mL of permeabilization buffer. Then cells were resuspended in 100 µL of permeabilization buffer and incubated with Ki67 antibody for 30 min at room temperature, washed twice with 2mL of permeabilization buffer and resuspended in 250 µL of PBS for data acquisition. Five-colour acquisition was performed on Cytomics FC 500 flow cytometer (Beckman Coulter, Fullerton, CA). For each sample, a minimum of 30.000 CD4+ and 30.000 CD8+ events were acquired. Data analysis was performed using CXP software (Beckman Coulter, Fullerton, CA).
