# Supplementary material for: HCV coinfection contributes to HIV pathogenesis by increasing immune exhaustion in CD8 T-cells
Source: PLoS One. 2017 Mar 21;12(3):e0173943. doi: 10.1371/journal.pone.0173943 (PMC5360268; doi:10.1371/journal.pone.0173943)
Supplement: S1 Table — Complete list of all monoclonal antibodies and fluorochromes used in the study. (DOC) [file pone.0173943.s002.doc]

**S1 Table.**  Monoclonal antibodies and fluorochromes used in the study

| **Antibody** | **Fluorochrome** | **Clone** | **Provider** |
| --- | --- | --- | --- |
| CD4 | PerCP-Vio700 | VIT4 | Miltenyi Biotec |
| CD8 | ECD | Thy2D3 | Beckman Coulter |
| CD31 | FITC | WM59 | BD Biosciences |
| CD38 | FITC | T16 | Beckman Coulter |
| Tim3 | PE | 344823 | R&D Systems |
| Ki67 | PE | 20Raj1 | eBioscience |
| CD45RA | ECD | 2H4 | Beckman Coulter |
| CD57 | PE-CF594 | NH-1 | BD Biosciences |
| HLADR | PE-Cy5 | Immu357 | Beckman Coulter |
| CD95 | PE-Cy7 | DX2 | BD Biosciences |
| PD1/biotin | Streptavidin-PECy7 | J105 | eBioscience |
