# Supplementary material for: HCV coinfection contributes to HIV pathogenesis by increasing immune exhaustion in CD8 T-cells
Source: PLoS One. 2017 Mar 21;12(3):e0173943. doi: 10.1371/journal.pone.0173943 (PMC5360268; doi:10.1371/journal.pone.0173943)
Supplement: S2 Table — Only significant correlations are shown. (DOC) [file pone.0173943.s003.doc]

**S2 Table.** Pearson correlation coefficients (bivariate analysis) of different T-cell subsets with CD4 counts and with HIV pVL, in the whole population of patients. Only significant correlations are shown.

| **T-cell subset group** | **T-cell subset** | **CD4 count** | **p-value** | **HIV pVL** | **p-value** |
| --- | --- | --- | --- | --- | --- |
| **CD4 exhaustion** | CD4+PD1-Tim3+ | **-** | **-** | **0.241** | **0.024** |
| **CD4 subsets exhaustion** | CD4+31+RA-PD1-Tim3+ | **-** | **-** | **0.275** | **0.01** |
|  | CD4+31+RA+PD1-Tim3+ | **-** | **-** | **0.263** | **0.013** |
|  | CD4+31+RA+PD1+Tim3+ | **-0.282** | **0.008** | **0.226** | **0.034** |
|  | CD4+31-RA-PD1-Tim3+ | **-** | **-** | **0.279** | **0.008** |
|  | CD4+31-RA+PD1+Tim3+ | **-0.266** | **0.012** | **-** | **-** |
|  | CD4+31-RA+PD1+Tim3- | **-0.321** | **0.002** | **-** | **-** |
| **CD8 exhaustion** | CD8+PD1-Tim3+ | **-0.315** | **0.002** | **-** | **-** |
|  | CD8+PD1+Tim3+ | **-0.235** | **0.03** | **0.336** | **0.001** |
| **CD8 activation** | CD8+38-DR+ | **-** | **-** | **-0.307** | **0.003** |
|  | CD8+38+DR+ | **-0.333** | **0.001** | **0.333** | **0.001** |
|  | CD8+38+DR- | **-0.384** | **<0.0001** | **0.470** | **<0.0001** |
| **CD8 subsets exhaustion** | CD8+38-DR+PD1-Tim3+ | **-** | **-** | **0.241** | **0.023** |
|  | CD8+38-DR+PD1+Tim3+ | **-** | **-** | **0.314** | **0.003** |
|  | CD8+38+DR+PD1-Tim3+ | **-0.267** | **0.011** | **-** | **-** |
|  | CD8+38+DR+PD1+Tim3+ | **-0.251** | **0.017** | **0.312** | **0.003** |
|  | CD8+38+DR-PD1-Tim3+ | **-0.227** | **0.031** | **-** | **-** |
|  | CD8+38+DR-PD1+Tim3+ | **-** | **-** | **0.269** | **0.01** |
| **CD4**  **apoptosis/senescence** | CD4+95+57- | **-0.390** | **<0.0001** | **-** | **-** |
|  | CD4+95+57+ | **-0.223** | **0.034** | **-** | **-** |
|  | CD4+31+95+57- | **-0.309** | **0.003** | **0.230** | **0.03** |
|  | CD4+31-95+57- | **-0.518** | **<0.0001** | **-** | **-** |
| **CD4 subsets**  **turnover** | CD4+31+Ki67+ | **-0.221** | **0.036** | **-** | **-** |
|  | CD4+31-Ki67+ | **-0.397** | **<0.0001** | **0.346** | **0.001** |
| **CD4 subsets apoptosis/senescence** | CD4+31+Ki67-95+57- | **-0.291** | **0.005** | **0.224** | **0.033** |
|  | CD4+31+Ki67+95+57- | **-** | **-** | **0.406** | **<0.0001** |
|  | CD4+31-Ki67-95+57- | **-0.447** | **<0.0001** | **-** | **-** |
