# Supplementary figures and images for: HCV coinfection contributes to HIV pathogenesis by increasing immune exhaustion in CD8 T-cells
Source: PLoS One. 2017 Mar 21;12(3):e0173943. doi: 10.1371/journal.pone.0173943 (PMC5360268; doi:10.1371/journal.pone.0173943)

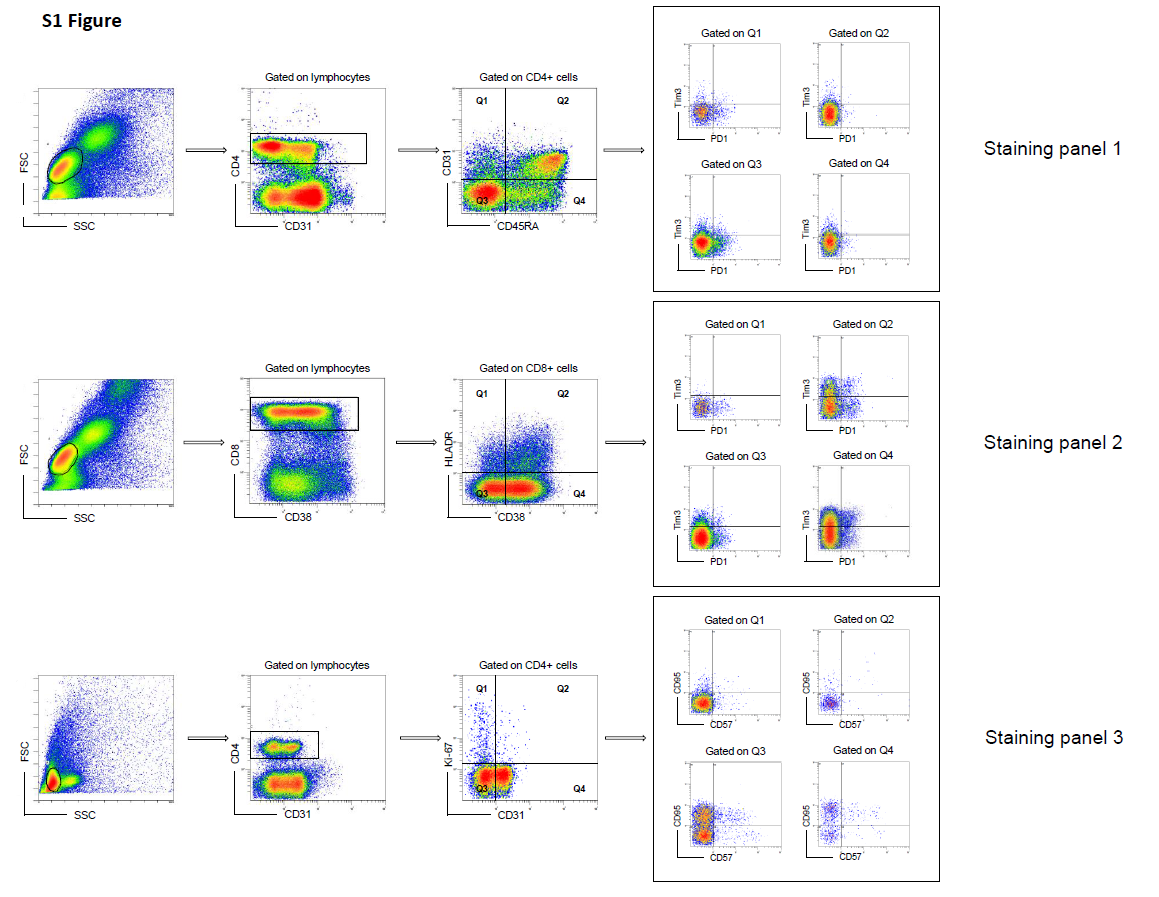

Supplement: S1 Fig — An initial gating was applied using forward (FSC) and side (SSC) scatter. From the population of lymphocytes a gate was placed to select CD4+ T-cells (staining panels 1 and 3) or CD8+ T-cells (staining panel 2). Then, the coexpression of CD45RA and CD31 and the coexpression of CD31 and Ki-67 were analyzed in CD4 T-cells and the coexpression of CD38 and HLADR was analyzed in CD8 T-cells. Lastly, expression of exhaustion markers PD1 and Tim3 was analyzed in different subsets of CD4 and CD8 T-cells, and the expression of CD95 and CD57 was analyzed in different subsets of CD4 T-cells. (TIF) [file pone.0173943.s004.tif]
